# Case Report: *Mycobacterium kansasii* causing infective endocarditis explored by metagenomic next-generation sequencing

**DOI:** 10.3389/fcimb.2023.1227537

**Published:** 2023-08-22

**Authors:** Liuqing Yang, Ling Peng, Ke Yuan, Kanru Cai, Cheng Feng, Gendong Yang, Shunyao Wang, Xiuyun Zhu, Jieyun Zhang, Fuxiang Wang, Hongzhou Lu

**Affiliations:** ^1^ The Third People’s Hospital of Shenzhen, The Second Affiliated Hospital of Southern University of Science and Technology, Shenzhen, China; ^2^ BGI Genomics, BGI Shenzhen, Shenzhen, China

**Keywords:** nontuberculous mycobacteria (NTM), *Mycobacterium kansasii*, infective endocarditis, metagenomic next-generation sequencing (mNGS), CNV (copy number variant)

## Abstract

In this report, we describe the first case of infective endocarditis caused by *Mycobacterium kansasii* in a 45-year-old male patient who presented with a 10-day fever and decompensated cirrhosis. Despite negative results in blood culture and pathology, we employed metagenomic next-generation sequencing (mNGS) to analyze the genome sequences of both the host and microbe. The copy number variation (CNV) indicated a high risk of liver disease in the patient, which correlated with biochemical examination findings. Notably, *M. kansasii* sequences were detected in peripheral blood samples and confirmed through Sanger sequencing. Unfortunately, the patient’s condition deteriorated, leading to his demise prior to heart surgery. Nevertheless, we propose that mNGS could be a novel approach for diagnosing *M. kansasii* infection, particularly in cases where blood culture and pathology results are unavailable. It is important to consider *M. kansasii* infection as a potential cause of endocarditis and initiate appropriate anti-infection treatment.

## Introduction

Non-tuberculous mycobacteria (NTM) encompass a group of *Mycobacterium* species, excluding the *Mycobacterium tuberculosis* complex and *Mycobacterium leprea*, which are widely found in nature (Johansen et al., 2020; Zhou et al., 2020). NTM has the potential to cause damage to various organs, including lung tissue, lymph nodes, skin, joints, and other organs (Margaret and John, 2014). These pathogens primarily affect individuals with weakened immune systems, particularly the elderly who may have underlying predisposing conditions (Tan et al., 2021). In the clinic, it is difficult to distinguish NTM infection from tuberculosis, which adds difficulties to clinical treatment and prevention. NTM has become an important health problem endangering human health (Kobashi et al., 2018). The incidence and prevalence of NTM are increasing in mainland China year by year, and the positive rate of NTM is higher in the southern coastal areas of China especially in Guangdong province (Liu and Song, 2021). Here, we present a case study of a patient diagnosed with infective endocarditis caused by *M. kansasii.* The identification of the pathogens DNA in the patient’s blood and copy number variation (CNV) analysis in the host’s genomic sequence was accomplished using metagenomic next-generation sequencing (mNGS).

## Case

**Figure 1:**
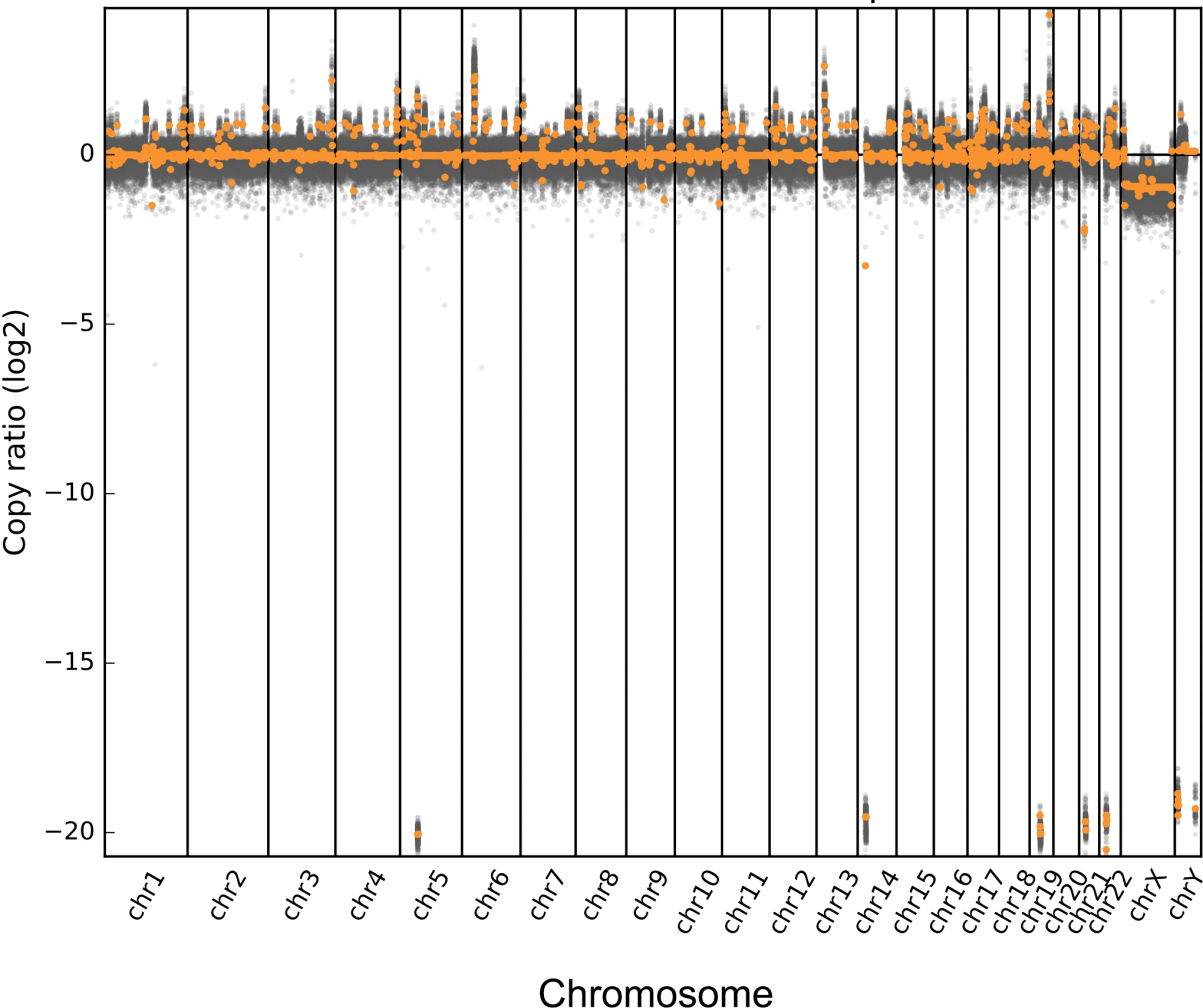
Copies of number variation data derived from peripheral blood from the patients. The horizontal axis represents the position of the chromosome, and the vertical axis represents the log2 value of the copy number. Gray dots represent each sliding window interval (bin), while orange dots represent each segment.

A 45-year-old male patient presented with a 10-day history of high fever reaching 40°C, along with symptoms of chills, excessive sweating, significant fatigue, and left abdominal pain. He did not have symptoms of headache, vomiting, cough, expectoration, chest tightness, or shortness of breath. Before admission, the patient had received imipenem and teicoplanin for antimicrobial treatment, but he continued to experience persistent high fever and chills. As a result, he was subsequently transferred to specialized infectious disease hospital for further evaluation and treatment. Laboratory results of complete blood count showed leukopenia and mild anemia. Specifically, the white blood cell, red blood cell, hemoglobin, and platelets were 3.42×10^12^/L, 93 g/L, 286×10^9^/L, respectively. The CNV analysis through host sequence suggests high-risk liver problems (as shown in **Figure 1**). Blood biochemical examination showed an increased serum level of Lactate Dehydrogenase (398U/L) and decreased serum levels of cholinesterase (1,640 U/L), while triglycerides were not significantly elevated (2.41 mmol/L). In the antigen test, HBsAg, HBeAb, and HBcAb showed a positive result, but the PCR test was negative for HBV DNA.

**Figure 2:**
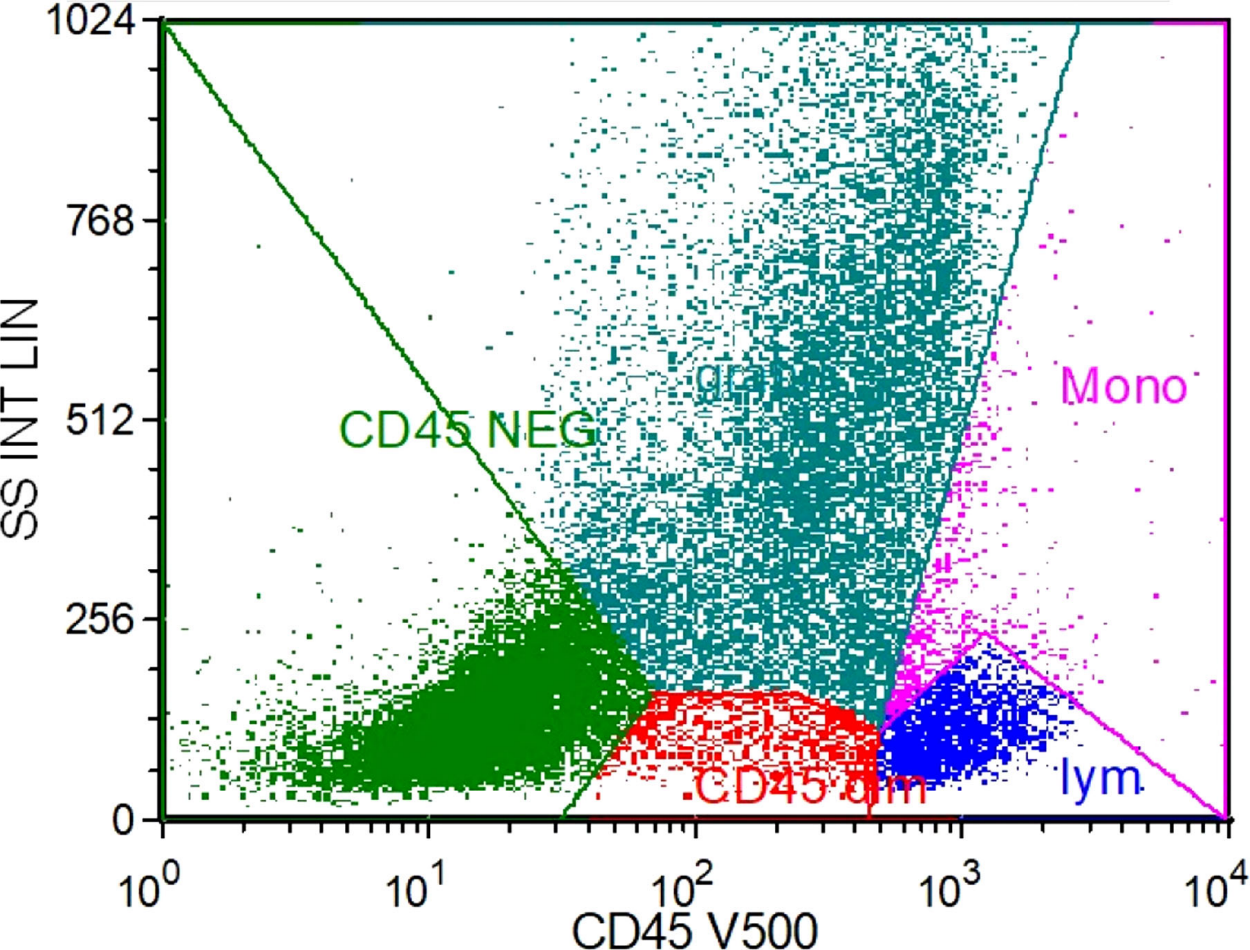
Flow cytometry immunofluorescence analysis results. Y-axis: lateral scattering intensity linear (SS INT LIN); X-axis: the account of CD45 detected by optical filter centered near 500 nm. The dark green color represents granulocyte, the purple color represents monocytes, the red color represents weak expressing CD45 cells, and the light green represents CD45 negative cells.

**Figure 3:**
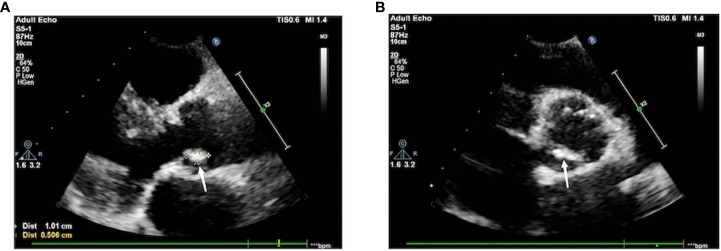
Cardiac color Doppler ultrasound. There is a slightly echogenic mass measuring approximately 10 mm × 5mm on the aortic valve, with low mobility. **(A)** The long-axis view of the left ventricle next to the sternum shows an attached vegetation on the aortic valve without coronary valve involvement. **(B)** The short-axis view of the left ventricle next to the sternum shows the formation of a vegetation on the aortic valve without coronary valve involvement. A small amount of fluid-filled dark area is visible in the pericardial cavity, with an anterior–posterior diameter of 3 mm.

**Figure 4:**
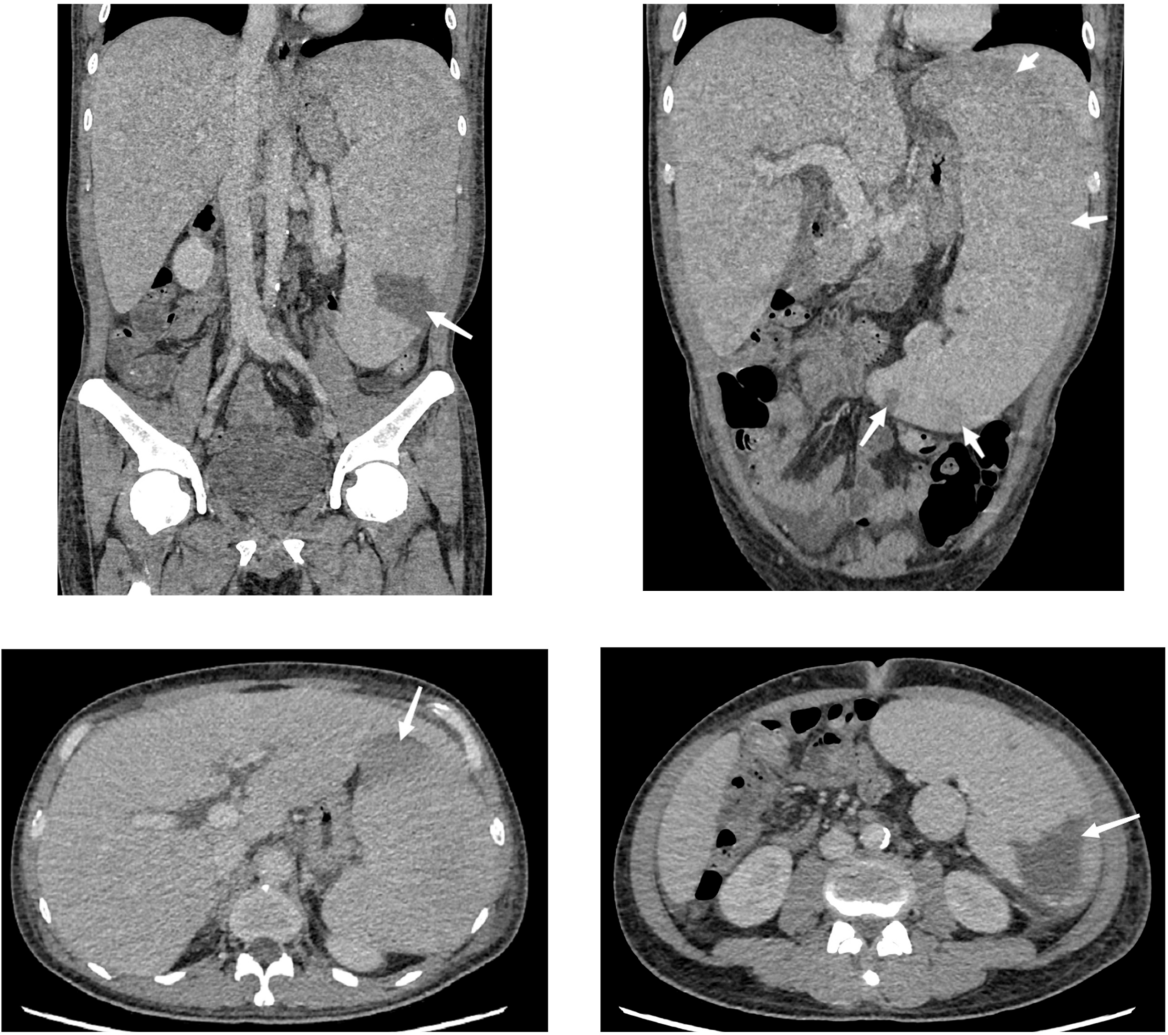
Abdominal contrast-enhanced computed tomography cross-section. The liver is fully voluminous and has a uniform density. The spleen is significantly enlarged and thickened, with the thickest part measuring approximately 82 mm. The edges are rounded, and the lower border of the spleen is below the lower border of the liver. There are multiple hypointense or non-enhancing wedge-shaped areas near the edge of the spleen, suggesting splenic infarction (arrows). A nodular accessory spleen is also observed along the inner edge of the spleen.

The bone marrow cytology results showed a decrease in bone marrow hyperplasia and a normal proportion of granulocytes. However, there was a significant increase in eosinophils and erythrocytes. Flow cytometry analysis of the bone marrow showed that T cells accounted for 88.3% of lymphocytes, NK cells accounted for 2.4%, and B cells accounted for 2.7%. The CD4:CD8 ratio was 1.20, indicating no significant immune abnormalities (**Figure 2**). Echocardiograms were performed on the second and eighth day after admission, revealing vegetation measuring 10×5 mm on the margin of the aortic valve, specifically the non-coronary valve (**Figure 3**). An ultrasound examination of superficial lymph nodes throughout the body showed mild enlargement of the left supraclavicular lymph node (measuring 12 mm ×9 mm and 9 mm ×5 mm). The liver and spleen exhibited marked enlargement. Furthermore, multiple wedge-shaped areas near the edge of the spleen parenchyma, appearing as hypo-enhancing or non-enhancing regions, were indicative of splenic infarction (**Figure 4**).

Following negative results from multiple blood and bone marrow cultures, the patient’s blood was subjected to mNGS on three separate occasions. Every time, mNGS revealed the presence of *M. kansasii* as indicated in **Table 1**. The data that support the findings of this study have been deposited into CNGB Sequence Archive (CNSA) (Chen et al., 2020; Guo et al., 2020). To validate the findings of mNGS, two pairs of primers were designed to amplify rpoB gene fragments, which were subsequently sequenced by a third-party company. Both sequences confirmed the presence of *M. kansasii*, aligning with the results obtained from mNGS as depicted in **Supplementary Figures S1**, **S2**. From the above results, we diagnosed the patient with *M. kansasii* endocarditis, decompensated liver cirrhosis (hepatitis B and alcohol-related), splenic infarction, peritonitis, and agranulocytosis. The rifampicin, ethambutol, azithromycin, and isoniazid were administered simultaneously for anti-NTM treatment. Other auxiliary treatments included supplementing albumin, human granulocyte-stimulating factor, squaganol, polysaccharide iron, and folic acid to support the patient. Despite these efforts, the patient continued to have recurrent fever. Subsequently, the patient’s family transferred him to Guangdong People’s Hospital for heart surgery, but unfortunately, the patient died in their ICU 1 week after discharge; thus, the heart surgery could not be performed.

**Table 1:** Results of mNGS in blood of patient.

Genus Type	Name	Sequence number	Species name	Sequence number	Relative abundance
G+	*Mycobacterium*	13	*Mycobacterium kansasii*	12	1.13%
G+	*Mycobacterium*	15	*Mycobacterium kansasii*	5	0.87%
G+	*Mycobacterium*	6	*Mycobacterium kansasii*	5	2.77%

## Discussion

Typically, infective endocarditis cases are attributed to pathogens like *Mycobacterium avium*, *Mycobacterium fortuitum*, *Mycobacterium chelonae*, *Mycobacterium abscessus*, *Mycobacterium neoaurum*, among others (Jagadeesan et al., 2013; Van Ingen, 2017). However, this is the first documented instance of native valve endocarditis caused by *M. kansasii*. Despite repeated negative cultures of the patient’s blood and bone marrow, which precluded invasive tissue cultures, the presence of *M. kansasii* DNA sequences was identified using mNGS, while no other pathogens were detected. Furthermore, the CNV analysis of the host sequence revealed a heightened risk of liver disease, consistent with imaging and biochemical examination findings. These findings underscore the valuable application of mNGS in exploring the host–microbe relationship.

While most cases of endocarditis occur following prosthetic valve or bioprosthetic valve placement, pacemaker implantation, or underlying rheumatic heart disease (Dalvi and Das, 2018; Bouchiat et al., 2020), it is important to note that the patient in this case did not have any of these underlying conditions. Furthermore, the flow cytometry results of the patient’s bone marrow indicated a normal immune state. The diagnostic process posed challenges in identifying the pathogen. However, on the third day of admission, the patient’s blood was subjected to mNGS, which successfully detected *M. kansasii* 48 h later. To validate the diagnosis, two additional mNGS tests were conducted, all of which confirmed the presence of *M. kansasii*. In conjunction with the identification of aortic valve vegetations through echocardiography on two separate occasions after admission, we were able to establish the diagnosis of *M. kansasii* endocarditis.

Following the 2020 “Guidelines for diagnosis and treatment of nontuberculous mycobacterial disease” and the IDSA-ATS guidelines (Branch CMAT, 2020), we initiated a combination therapy comprising intravenous rifampicin, isoniazid, oral ethambutol, and azithromycin for the patient. However, given the critical condition of the patient and the presence of hepatosplenomegaly, which could be attributed not only to hepatitis B virus and alcoholic-related decompensated cirrhosis but also to susceptibility to *M. kansasii* infection. Despite our efforts, the patient’s body temperature failed to normalize, and the overall condition did not improve. Unfortunately, the patient succumbed to the illness after being transferred to the hospital, indicating a lack of favorable response to the treatment. There are multiple factors contributing to the poor clinical efficacy of drug treatment in this case. First, the patient had pre-existing Child–Pugh C cirrhosis, which compromised liver function. Additionally, the delay from the definitive diagnosis of *M. kansasii* infection approximately 1 month resulted in severe condition. The presence of evident heart valve neoplasms indicates the severity of the disease and a high bacterial load, leading to a diminished response to anti-infective treatment. The patient’s rapid disease progression and the involvement of multiple organ failures such as liver dysfunction, agranulocytosis, lymphocyte deficiency, and septic shock highlight the challenging nature of the infection, making it difficult for anti-infection treatment to achieve timely rescue. Therefore, mNGS is an essential diagnostic method for non-tuberculous mycobacterium disease, especially when culture and pathology are not available. If the patient had been diagnosed before the growth of heart valve vegetation, the patient may have been more likely to survive.

## Data availability statement

The datasets presented in this study can be found in online repositories. The names of the repository/repositories and accession number(s) can be found below: https://db.cngb.org/mycngbdb/submissions/project, CNP0004591.

## Ethics statement

The studies involving humans were approved by the Third People’s Hospital of Shenzhen. The studies were conducted in accordance with the local legislation and institutional requirements. The participants provided their written informed consent to participate in this study. Written informed consent was obtained from the individual(s) for the publication of any potentially identifiable images or data included in this article. Written informed consent was obtained from the participant/patient(s) for thepublication of this case report.

## Author contributions

The authors contributed to this case report in the following ways. LY and FW were responsible for the conception and design of the report, acquisition, analysis, and interpretation of the patient’s clinical data, KY and LY drafted the initial manuscript. LP contributed to the literature review, provided critical revisions for intellectual content, and assisted in the finalization of the manuscript. KC and CF contributed to the collection and interpretation of laboratory and imaging data, and provided substantial input in revising the manuscript. HL provided expert guidance, supervised the project, reviewed and revised the manuscript for important intellectual content. GY, SW, and XZ ensured the accuracy and integrity of the report. All authors read and approved the final version of the manuscript and agree to be accountable for all aspects of the work presented.
