# Supplementary material for: Case Report: Mycobacterium kansasii causing infective endocarditis explored by metagenomic next-generation sequencing
Source: Front Cell Infect Microbiol. 2023 Aug 22;13:1227537. doi: 10.3389/fcimb.2023.1227537 (PMC10482420; doi:10.3389/fcimb.2023.1227537)

**Supplement Materials**

Method

Sample processing and DNA extraction

A blood volume of 3 ml was collected from patients, placed in a blood collection tube and stored at room temperature for 3-5 minutes before, within 8 hours of collection, the plasma was separated and centrifuged at 4000 rpm for 10 minutes at 4 °C. Plasma samples were transferred to new sterile tubes. DNA was extracted from 300 μl of plasma using the TIANamp Micro DNA Kit (DP316, TIANGEN BIOTECH, Beijing, China) according to the manufacturer's instructions. The extracted DNA samples were used to construct DNA libraries.

Construction and sequencing of the DNA library

The DNA libraries were then constructed by DNA fragmentation, end repair, adapter ligation and PCR amplification.Agilent 2100 was used for quality control of DNA libraries. High quality libraries were pooled, DNA Nanoball (DNB) was generated and sequenced on the MGISEQ-2000 platform.

Bioinformatics Analysis

High-quality sequencing data was generated by removing low-quality reads and then computationally subtracting human host sequences that were mapped to the human reference genome (hg19) using a Burrows-Wheeler alignment . The remaining data were classified by removing the low-complexity reads by concurrent matching against the Metagenomics Database of Pathogens (PMDB), which consists of bacteria, fungi, viruses, and parasites. The classification reference databases were downloaded from NCBI (ftp://ftp.ncbi.nlm.nih.gov/genomy/).

Sanger sequencing

In this experiment, The target bacterial DNA in the sample could distinguishable band in the figure S1. The identification of the band as belonging to *M. kansasii* was confirmed by DNA sequencing. The targeted DNA was performed with the sequenced nucleotide. It shows that the amplified nucleotide sequence of *M. kansasii* with highest score figure S2.

Figure S1: The PCR result of the RpoB fragment from serum sample. A represents experimental sample. B represents negative control.


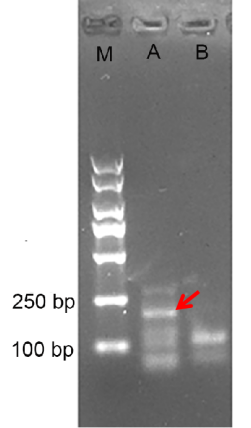


Figure S2: The NCBI nucleotide BLAST

The sequencing results are as follows:

TGAAGTGGTCGATGTCGTCCTGGGGCAGCGTCCAGTCGGCCCGGTCGGACTCGGTGTTCTCACCGTCGGCACGCTGACCTTCCGGCAGGGCTCCGACGAAGAAATAGGTGTCGTAACGACGGGTGAGCTCAGCCTCCGGGGTGACCCAATTGGCCCAGGGGCGCAGCAGGTCGTAGCGCTTCTCCTTGAAGAACAA


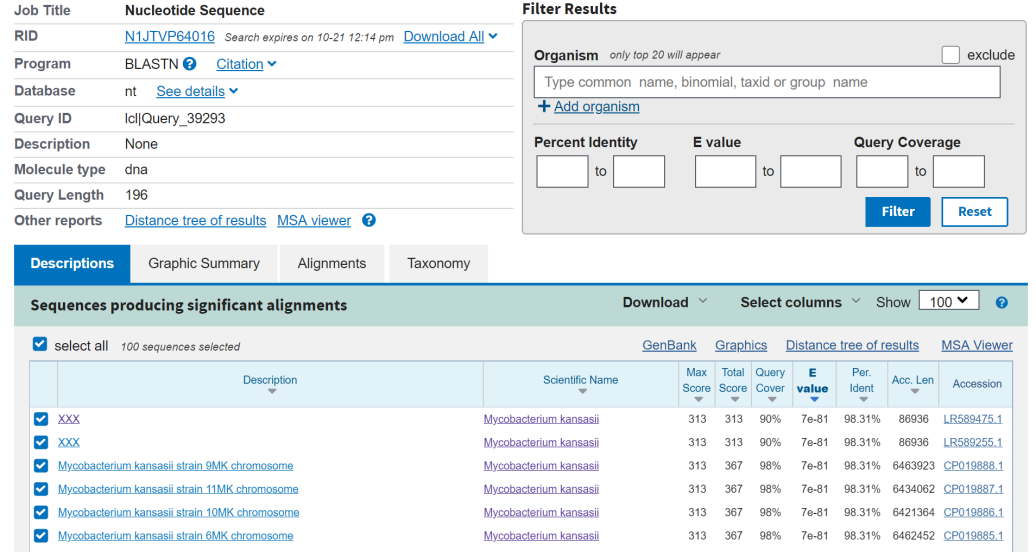

Supplement: Supplementary file 1 [file DataSheet_1.docx]
